# Dietary inclusion of fibrous corn silages reduces gastric mucosa damage in fattening heavy pigs

**DOI:** 10.1186/s40813-024-00391-9

**Published:** 2024-11-22

**Authors:** M. Spanghero, M. Braidot, M. Orioles, C. Sarnataro, I. Pividori, A. Romanzin

**Affiliations:** Department of Agricultural, Food, Environmental and Animal Sciences, University of Udine, Via Sondrio, 2/A, 33100 Udine, Italy

**Keywords:** Heavy pig, Corn silages, Gastritis, Hair steroids

## Abstract

**Background:**

Several surveys conducted at slaughter sites have highlighted that gastric lesions are a widespread issue in fattening pigs, mainly due to feeding regimes. Diets with small particle sizes and low fibre contents guarantee high digestibility and performance but generate more rapid stomach emptying with a negative effect on gastric mucosa integrity. Providing fattening pigs with fibrous materials (e.g., straw provided in racks) or coarse fibrous ingredients (e.g., coarse silages) reduced the presence of gastric ulcers. The present research compares a traditional corn-soy-based diet with an experimental diet where bran and a portion of corn meal was substituted with whole ear and whole plant corn silages at the maximum dosages permitted by new Protected Designation of Origin for Italian dry-cured ham (20 and 10% of DM, respectively). This study aimed to examine the impact of the inclusion of corn silages in the diet on the productive performance of heavy Italian pigs and their ability to mitigate gastric mucosa damage.

**Results:**

The growth performances were satisfactory (750–800 g/d) given the advanced interval of growth of animals (from 120 to 180 kg). However, the inclusion of corn silages tended to reduce the growth rate by 5–6% due to the reduction of organic matter digestibility, without compromising the slaughter traits or the back-fat fatty acid profile. The experimental diet substantially affected both stomach development and mucosal integrity. The first consequence was an increase in stomach weight of approximately 6% (*P* < 0.01) but the most notable advantage of coarse feeding was a reduction in stomach damage severity, with a low number of cases with higher scores in animals fed coarse materials (*P* < 0.01).

**Conclusions:**

The dietary inclusion of corn silages (30% of diet DM) decrease effectivelly the severity of stomach damage in finishing heavy pigs. Based on the feeding trial performances, the perspective of feeding heavy pigs corn silage should consider specific agronomic and harvesting techniques to improve digestibility and not reduce the growth rate.

**Supplementary Information:**

The online version contains supplementary material available at 10.1186/s40813-024-00391-9.

## Introduction

Several surveys conducted at slaughter sites have highlighted that the presence of gastric lesions is a widespread issue in fattening pigs. Swaby and Gregory [50] analyzed and scored approximately 10 thousand stomachs, obtaining an overall prevalence of gastric lesions of around 80%, with an incidence of 73% of ‘mild’ lesions. Cybulski et al. [5] examined 32 thousand stomachs from different farms and assessed gastric injuries in 72% of the total samples taken into account. Helbing et al. [23] reported that 61% of pigs (one thousand subjects controlled) showed lesions ranging from mild to severe with no improvements compared to a previous survey conducted in 2005 in Switzerland. In the Italian context, Gottardo et al. [21] documented that 21% of more than 20 thousand heavy pig stomachs controlled at slaughter presented mild or severe ulceration.

There are different factors (such as density, stress, and transport) that can be linked to the occurrence of gastric mucosa injury, but the feeding regime is the most important one. Recently, Cybulski et al. [6] reported that only a few nondietary risk variables were associated with the incidence of stomach damage in finishing pigs. In contrast, several studies have demonstrated that diets with small particle sizes and low fibre contents guarantee high digestibility and performance but generate more rapid stomach emptying with a negative effect on gastric mucosa integrity [36–38]. Exposure to low pH, caused by rapid gastric transit and hence a comparatively empty stomach, can lead to the development of parakeratosis in the *pars oesophagea* (OA) of the stomach, which commonly evolves through fissures, erosions, and ulcerations. The squamous epithelium of this region has no buffering capacity, thus making it highly susceptible to attack by increased gastric acidity [51]. Providing fattening pigs with fibrous materials (e.g., straw provided in racks) or coarse fibrous ingredients (e.g., coarse silages) reduced the presence of gastric ulcers [7, 17, 33].

In Italy, pigs are mainly slaughtered at a high body weight (BW) for cured ham production (carcass weight between 110 and 168 kg). In the late finishing phase (e.g., above 80 kg of BW), heavy pigs can digest fibrous feeds rather efficiently [18–20] and have a well-developed gut. Moreover, coarse fibrous materials could counteract rapid gastric emptying after a meal, protecting the stomach mucosa from gastric hydrochloric acid. Therefore, recent dietary rules for fattening heavy pigs in Italy for the production of Protected Designation of Origin (PDO) dry-cured ham [12] authorize the use of corn silages, such as whole ear and whole plant corn silages (WECS and WPCS, respectively). The scientific interest in feeding practices for heavy pigs should be wide and not limited to the areas of heavy pig production (mainly Italy but also Spain and the Balkan countries) because there is an overall tendency to increase pig weight at slaughter. In the United States and Canada, pig slaughter weight has been progressively growing without affecting meat quality [43, 55], and the expected increase will generate carcasses weighing approximately 118 kg by 2050.

The present research compares a traditional corn-soy-based diet (CTR) with a diet in which bran and a portion of corn meal were substituted with WECS and WPCS (SIL) at the maximum dosages permitted by new PDO constraints (20 and 10% of DM, respectively). The aim of this study was to examine the impact of the inclusion of corn silage in the diet on the productive performance of heavy Italian pigs and its ability to mitigate gastric mucosa damage.

## Materials and methods

### Feeding trial and digestibility measures

The feeding trial was divided into two successive identical experimental periods (blocks) of 70 days each. In each period, 18 “Italian Large White × Italian Landrace” barrows (about seven months of age and 128 kg BW) were divided into six groups of three pigs that were homogeneous for BW and kept in six pens (3 × 3 m, partially slatted). During the first experimental period, pens were randomly assigned to CTR and SIL diets (three pens/dietary treatment), and the same assignment was followed during the second period to test each dietary treatment in six pens throughout the trial.

The CTR diet contained corn meal, barley meal, wheat bran, supplement and brewer yeast, while in the SIL diet, part of the corn (21% dry matter, DM) and all the wheat bran were substituted with WECS and WPCS (20 and 10% DM), as detailed in Table 1. The particle size of the two ingredients was assessed by fractionating a fresh sample of silages with the Penn State Particle Separator (mesh diameter 19, 8, and 1.18 mm) as reported by Kononoff et al. [27]. Considering particle distribution, an average dimension of 5.73 and 9.44 mm were calculated for WECS and WPCS respectively. The two diets were equalized in terms of crude protein (CP) by increasing the amount of soybean meal from 90 to 120 g/kg in the SIL diet. The CTR diet was prepared at the beginning of each feeding phase whereas the SIL diet was prepared daily by mixing WECS and WPCS with the other pre-mixed ingredients.

**Table 1:** Ingredient composition and chemical analysis of the experimental diets

	DIETS^1^		WPCS^2^	WECS^3^
	CTR	SIL		
Ingredient, g/kg DM				
Whole ear corn silage	0	200		
Whole plant corn silage	0	100		
Corn meal	600	390		
Barley meal	160	160		
Soya bean meal, extracted	90	120		
Wheat bran	120	0		
Supplement ^4^	25	25		
Brewer yeast	5	5		
Chemical analysis				
DM, %	87.1	75.9	40.5	51.5
CP, % DM	14.3	14.0	7.28	7.83
NDF, % DM	17.9	18.1	34.9	23.5
ADF, % DM	5.4	7.3	18.1	9.86
EE, % DM	3.5	2.9	2.75	2.78
Ash, % DM	5.1	5.1	3.72	1.74
Ca, % DM^5^	0.66	0.70		
P, % DM^5^	0.49	0.36		
Lys, % DM^5^	0.81	0.84		

NDF = Neutral detergent fiber; ADF = Acid detergent fiber. EE = Ether extract

^1^ CTR = Control diet with 0% corn silages; SIL = Diet containing whole ear corn silage and whole plant corn silage (20 and 10% on a DM basis, respectively)

^2^ WPCS = Whole plant corn silage

^3^ WECS = Whole ear corn silage

^4^ Contents per kilogram of supplement: vitamin A 216000 IU; vitamin D_3_ 45,000 IU; vitamin E 800 mg; vitamin K_3_ 50 mg; vitamin B_1_ 100 mg; vitamin B_2_ 100 mg; Ca 225 g; Na 59 g; P 16 g; Mg 13.5 g; Lys 85 g

^5^ Calculated values based on the NRC [40] tables of composition and the supplement contents

Each pen was equipped with a faucet for drinking water and three separate troughs to avoid competition for feeding. The daily DM intake (DMI) was restricted to 77.1 g/kg BW^0.75^, and rations were manually fed in equal portions at 08:00 and 16:00. The trial started with an initial BW of 128 ± 6.9 kg. The weight of the pigs and the average daily gain (ADG) were monitored at the halfway point and at the end of the trial. The BW and pen feed intake of the pigs were used to calculate the ADG, average daily feed intake (ADFI), and feed-to-gain ratio (F:G). During the initial and final weightings, hair samples were obtained from each animal.

The feed samples were periodically collected for proximate analysis, and the digestibility of the diets was determined using acid-insoluble ash (AIA) as a marker. To increase the content of AIA in the faeces, SiO_2_ (precipitated and dried: 95%) was added to the diets (2 g/kg) for 10 days before sampling. For three consecutive days, two samples of faeces were collected daily from each pen and were immediately stored at − 20 °C for subsequent analyses.

### Slaughtering traits and stomach measurements

The animals were slaughtered in a commercial slaughterhouse at an average BW of 183.0 kg (± 7.3 kg) by electrical stunning and exsanguination. The hot carcasses were weighed and then dissected into commercial cuts. Before cooling, the hams and loins were weighed, the back fat thickness was measured at the central line of the carcass between the third and fourth ribs [11], and a back fat sample (100–150 g) was collected for analysis of the fatty acid profile. The meat pH was measured on muscle sections by a glass piercing electrode (Crison 52—32) connected to a pH meter at 45 and 240 min after slaughter.

Samples of cecum content (approx. 50 g) were collected from each pig at slaughter, transferred to the laboratory and immediately frozen at − 20 °C for subsequent use in bacterial DNA analysis.

The stomachs were collected at slaughter, transferred to the laboratory, immediately opened along the greater curvature (*curvatura ventriculi major*), emptied, and gently rinsed.

**Fig. 1:**
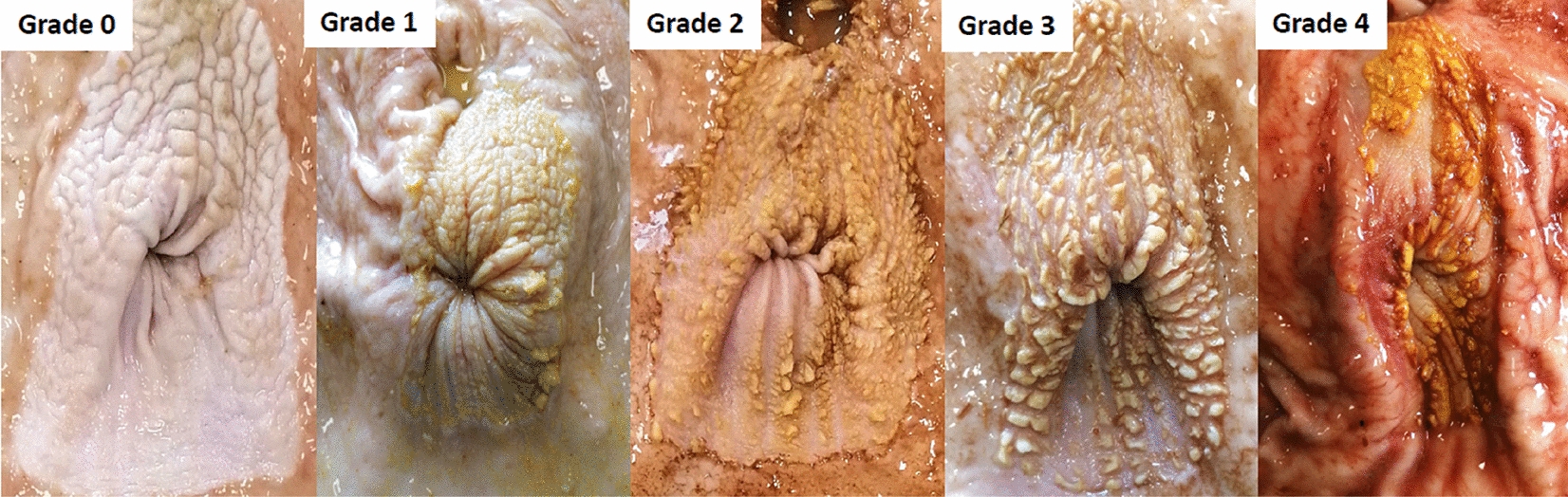
Gravity ulceration scale adopted for the evaluation of *pars oesophageal* of the stomach. Grade 4 represents the highest severity

The OA of the stomach wall was macroscopically evaluated using an adapted scale derived from earlier research [24, 28], with values ranging from 0 to 4, as shown in Fig. 1. The assigned score increased with wider proportions of the OA exhibiting parakeratosis, considering factors such as its extension, the presence of fissures and erosions, ulcerations, and re-epithelialization with contraction of the tissue. The organ was then weighed, and orthogonal photos of the outstretched stomach were taken. The images were used to measure the oesophageal, fundic, cardiac, and pyloric areas by ImageJ open-source software (v1.46r, [45]).

### Chemical analysis of feeds and faeces

Corn silage samples and faecal samples were dried for 48 h at 60 °C in a forced-air oven and then milled through a 1 mm sieve (Wiley mill, Arthur H. Thomas, Philadelphia, PA). The dry silages and compound feeds were assayed in duplicate for DM, CP (nitrogen N × 6.25), ether extract (EE), total ash content [1], methods 930.15, 976.05, 954.02 and 942.05, respectively), and AIA [26]. The AIA content in the dried feed and faecal samples was determined by slowly boiling the ash from the samples for 15 min in 75 mL of 3 N HCl [26], with only minor modifications). The solution was then filtered through ash-free filter paper (Whatman no. 541, 20 µm of porosity), and the filters with residues were incinerated at 550 °C for 1 night.

Neutral detergent fibre (NDF) and acid detergent fibre (ADF) contents were measured by an Ankom II fibre analyzer (Ankom Technology Corporation, Fairport, NY) following the procedure of Mertens [34].

The organic matter, NDF and CP coefficients of apparent total tract digestibility were calculated as follows on a DM basis: 100 − (100 × (% AIA in feed/% AIA in faeces) × (Fecal nutrient/% Feed nutrient)).

### Fatty acid profiles of backfat samples

Lipids were extracted from backfat samples according to the methanol–chloroform method of Folch et al. [15], and fatty acid methyl esters (FAME) were obtained using methanolic HCl via trans-esterification of triglycerides by following the method described by Sukhija and Palmquist [49]. Then, FAME were separated by gas chromatography-mass spectrometry (GC/MS) analyses, which were performed in EI mode (70 eV) with a 5977E MSD system (Agilent Technologies, Santa Clara, CA, USA), a 7820A GC system and a 7693A autosampler and automatic split/splitless injector. GC/MS analyses in full scan mode (m/z 50–600) were performed after a solvent delay of 7 min at 3 microscan/s. Compounds were identified by comparing their mass spectra with spectra from the National Institute of Standards and Technologies (NIST) Mass Spectral Library; the FAMEs were quantified using C19:0 as the internal standard and were expressed as the percentage of the total fatty acids.

### 16S sequencing and bioinformatics analysis

Samples of cecum contents were thawed and the bacterial DNA was extracted using the QIAsymphony DSP Virus/Pathogen Kit according to the manufacturer’s instructions. DNA samples were quantified by using a Qubit 2.0 fluorometer (Invitrogen) and a high-sensitivity DNA (HS) assay (Invitrogen). A total of 5 ng was used for library preparation via 16S metagenomic sequencing library preparation following Illumina’s instructions. Briefly, the v3 and v4 regions of the 16S ribosomal gene were selectively amplified, and unique sample indices were incorporated into each sample. Libraries were quantitated by a Qubit 2.0 fluorometer (Invitrogen) using a high-sensitivity DNA assay kit, and a Bioanalyzer 2000 (Agilent) high-sensitivity assay was used to determine the expected size distribution of the library fragments. Single libraries were pooled and then sequenced in paired-end 250 bp mode on an Illumina MiSeq instrument (Illumina).

An average of 100 k reads were sequenced and analyzed per sample. FASTQ files were uploaded into the BaseSpace tool for use with the 16S metagenomics app. Here, the raw data were processed for format conversion, sample demultiplexing, and microbiota composition analysis. Fragments were mapped to the RefSeq database for differential microorganism characterization. Alpha (Shannon and Chao1) and beta (Jaccard similarity) diversity indices were determined using the R environment (v 4.1.3, [44]) with the appropriate function of the Vegan package (v. 2.5–7; [41]).

### Hair cortisol and DHEA(S) analysis

Hair was collected from the back at the level of the last rib and approximately 10 cm along the side of the vertebral column; this area was chosen because of its cleanliness. Animals were shaved as close as possible to the skin with an electric razor designed for large animals, and samples were kept in paper envelopes in the dark at room temperature until analysis. Washing hair samples with isopropanol is essential to minimize the risk of extracting steroids from the surface of the hair, which are deposited by sweat and sebum. Cortisol and dehydroepiandrosterone (sulphate) (DHEA(S)) were extracted as described by Bergamin et al. [3]. Their concentrations were measured using an in-house enzyme-linked immunosorbent assay (ELISA), as described previously for human hair by Falco et al. [13].

### Statistical analysis

During the study, two pigs, one from each of the two dietary groups, were culled because they had severe health conditions and showed poor gain. The in vivo performance, digestibility coefficient, slaughter and stomach trait data were analyzed as a completely randomized 2 × 2 factorial design with SAS software (v9.4, SAS [48]) using the GLM procedure and considering the pen as the experimental unit:

$$y_{ijk} = \, \mu + \, \alpha_{i} + \, \beta_{j} + \, \varepsilon_{ijk}$$

where y _ijk_ is the response of the experimental pen (k = 1,3), μ is the overall mean, αi is a fixed effect of the type of diet (i = 1,2), β_j_ is a random effect (block) of the experimental period (j = 1,2), and ε _ijk_ is the random error. The levels of the cortisol and DHEA hormones in the hair of the pigs and their ratios were statistically analyzed using the GLM procedure as a factorial randomized complete block (experimental phase) design with repeated measures as follows:

$$y_{ijkm} = \, \mu + \, \alpha_{i} + \, \beta_{j} + \, \gamma_{m} + \, \left( {\gamma \, \times \, \alpha } \right)_{mi} +\varepsilon_{ijkm}$$

where y _ijkm_ is the response of the experimental pen (k = 1,3), μ is the overall mean, α_i_ is a fixed effect of the type of diet (i = 1,2), β_j_ is a random effect (block) of the experimental period (j = 1,2), γ_m_ is the sampling time (m = 1,2), (γ ˟ α)_mi_ is the random effect of the interaction between diets and sampling time, and ε_ijkm_ is the random error.

The results of the gastric lesion score were examined by SAS software's PROC FREQ, which used the Fisher option for contingency table analysis with the individual pig as the experimental unit.

## Results

The chemical analysis provided in Table 1 shows that the diets used in the current research were designed with equal quantities of CP and NDF. The sole difference between the two diets was the ADF level, which was greater in the SIL diet (7.3 vs*.* 5.4% DM).

At the beginning of the trial (Table 2), the two dietary groups had similar BW, and no significant difference between the two groups was observed for the middle and final BW. Comparable DMI values were observed for both dietary groups, but the inclusion of silages tended to decrease the ADG (0.801 vs. 0.752 g/d) and G:F ratio (0.278 vs. 0.261 g/g DM) (*P* = 0.078 and 0.086, respectively). The SIL diet resulted in a significant decrease (*P* < 0.01) in OM and NDF digestibility (82.41 vs. 79.75% and 55.88 vs. 42.64%, respectively), whereas CP digestibility remained unchanged. The faeces of pigs fed the SIL diet had a lower DM content (23.31 vs. 27.90, *P* < 0.01, RMSE 1.42, data not in tables).

**Table 2:** Growth performance, coefficient of digestibility and slaughter traits of the pigs

	DIETS^1^		RMSE	*P* value
	CTR	SIL		
Initial BW, kg	127.7	127.9	7.92	0.953
Middle BW, kg	155.1	153.8	9.38	0.816
Final BW, kg	185.0	181.7	7.45	0.467
DMI, g/(kg BW^.75^)	65.20	65.57	2.57	0.814
ADG, g/d	0.801	0.752	0.05	0.078
G:F, g/g DM	0.278	0.261	0.01	0.086
OM digestibility, %	85.23	81.93	1.88	< 0.01
NDF digestibility, %	55.88	42.64	6.75	< 0.01
CP digestibility, %	78.06	77.76	2.87	0.770
Slaughter traits				
Carcass, kg	154.4	151.9	7.89	0.597
Dressing out, %	83.45	83.56	1.34	0.892
Ham weight, kg	19.74	19.54	1.23	0.784
Loin weight, kg	9.13	9.01	0.79	0.797
Back fat thickness, mm	30.11	29.25	1.34	0.305
pH_45_	6.00	6.11	0.21	0.408
pH_240_	5.49	5.48	0.09	0.903

DMI = Dry matter intake; ADG = Average daily gain; G:F = ADG/DMI;

^1^ CTR = Control diet with 0% corn silages; SIL = Diet containing whole ear corn silage and whole plant corn silage (20 and 10% on a DM basis, respectively)

Dietary treatments did not affect the main slaughter traits, with similar carcass weights and dressing proportions, comparable weights of ham and loin, and similar thicknesses of the back fat. Table 3 shows the fatty acid composition of the back fat between the two groups, and the only difference was the lower ω-6/ω-3 ratio for the SIL diet (20.78 vs. 22.29, *P* < 0.05).

**Table 3:** Fatty acid composition of the pigs’ back fat

	DIETS^1^			
	CTR	SIL	RMSE	*P* value
Fatty acid^2^, %				
C12:0	0.07	0.06	0.01	0.798
C14:0	1.27	1.25	0.06	0.969
C16:0	24.48	24.54	0.57	0.867
C16:1	2.01	2.07	0.13	0.976
C17:0	0.33	0.34	0.07	0.792
C18:0	14.16	14.70	0.96	0.362
C18:1 ω-9	39.92	39.68	1.18	0.735
C18:1 ω-7	2.22	2.29	0.15	0.416
C18:2 ω-6	12.33	11.52	1.09	0.243
C18:3 ω-3	0.49	0.48	0.06	0.980
ω-3	0.59	0.60	0.07	0.939
ω-6	12.91	12.37	1.32	0.502
ω-6/ω-3	22.29	20.78	0.76	0.011
Fatty acid profile^3^, %				
SFA	40.56	41.42	1.26	0.277
MUFA	45.63	45.60	1.44	0.971
PUFA	13.49	12.97	1.40	0.540

^1^CTR = Control diet with 0% corn silages; SIL = diet containing whole ear corn silage and whole plant corn silage (20 and 10% on a DM basis, respectively)

^2^Percentage of total determined fatty acid

^3^ SFA = Saturated fatty acids; MUFA = Monounsaturated fatty acids; PUFA = Polyunsaturated fats

Table 4 displays the principal stomach characteristics evaluated postmortem. The empty stomach weight was greater for subjects fed the SIL diet (1222 vs. 1106 g, *P* < 0.01), while the total stomach area exhibited similar values between the two diets. Nevertheless, when evaluating the extent of different stomach areas, a significant increase (*P* < 0.01) was observed in the OA (5.63 vs. 4.60%), and a significant decrease (*P* < 0.01) was noted for the fundic region (46.23 vs. 50.19%) in animals fed the SIL diet.

**Table 4:** Stomach weight and surface area of different regions

	DIETS^1^		RMSE	*P* value
	CTR	SIL		
Stomach traits				
Empty weight, g	1106	1222	56.09	< 0.01
Total area, cm^2^	895.0	934.2	87.00	0.461
Density, g/cm^2^	1.24	1.31	0.10	0.254
Stomach areas, % of total				
Oesophageal	4.60	5.63	0.51	< 0.01
Cardiac	24.57	24.70	1.87	0.730
Fundic	50.19	46.23	1.74	< 0.01
Pyloric	20.99	23.16	2.28	0.143

^1^ CTR = Control diet with 0% corn silages; SIL = Diet containing whole ear corn silage and whole plant corn silage (20 and 10% on a DM basis, respectively)

**Fig. 2:**
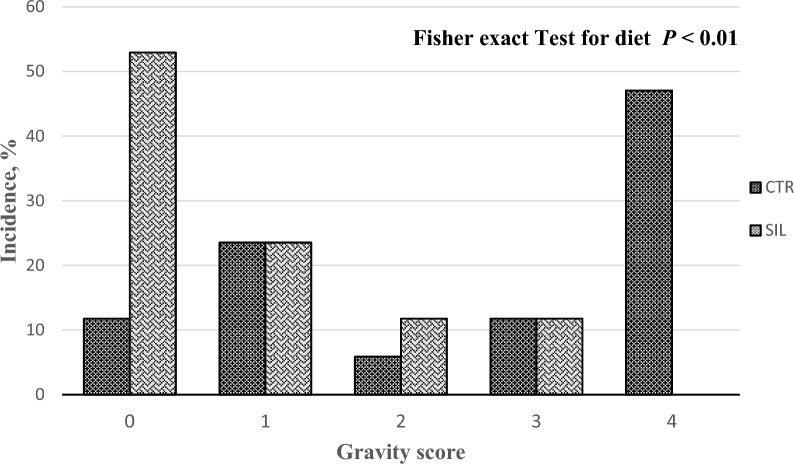
The incidence of gastric lesions in *pars oesophageal* divided by gravity score (highest severity: grade 4) for control diet (CTR) and diet with silages (SIL)

The score of gastric lesions present in the OA was affected by the dietary treatment (*P* < 0.01), and the SIL diet reduced the severity of gastric lesions (Fig. 2).

Similar levels of the hormones cortisol and DHEA (S) were detected in both groups at the beginning of the feeding trial. At the end of the dietary test, no significant variation in hormones was reached, with comparable values observed in cortisol and DHEA(S) values (Table 5).

**Table 5:** Cortisol and dehydroepiandrosterone (DHEA) concentrations in the hair of the pigs collected at the beginning and at the end of the fattening trial

	DIETS^1^				RMSE	*P* values		
	CTR		SIL			Diet	Sampling	Sampling*diet
	Initial	Final	Initial	Final				
Cortisol, pg/mg	5.19	1.98	6.99	2.48	2.42	0.260	< 0.01	0.520
DHEA(S), pg/mg	22.9	22.1	26.4	27.1	9.48	0.289	0.984	0.859
Ratio^2^, %	21.3	9.64	24.6	8.47	4.98	0.611	< 0.01	0.288

^1^CTR = Control diet with 0% corn silages; SIL = Diet containing whole ear corn silage and whole plant corn silage (20 and 10% on a DM basis, respectively)

^2^Ratio = Cortisol /DHEA(S)*100

**Fig. 3:**
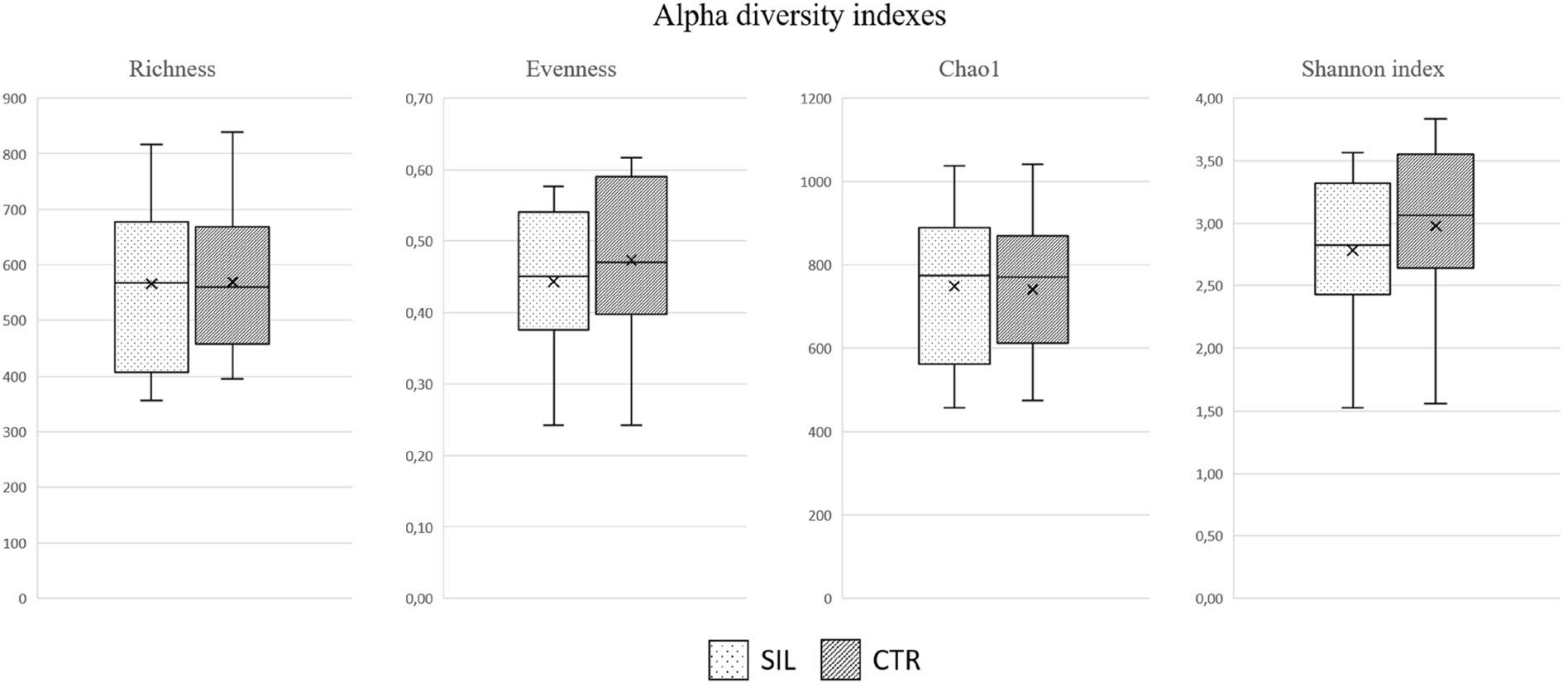
The alpha diversity indices calculated for the gut microbiota of animals fed with the control diet (CTR) or the diet containing silage (SIL)

16S DNA sequencing was performed to assess whether dietary treatments impact the cecal microbiota, affecting animal performance and nutrient digestibility. Each sample was analysed, yielding 248,941 readings in total. Dietary treatment had no significant effect on species richness or evenness, with comparable values for both groups (Fig. 3). Alpha diversity was calculated for all samples at the genus level: the Chao1 index (748.5 ± 187.8 and 740.8 ± 164.4 for CTR and SIL, respectively) and Shannon index (2.78 ± 0.62 and 2.98 ± 0.69 for CTR and SIL, respectively) were found to be similar in both dietary treatments, indicating similar microbiome diversity. When comparing the two groups at the temporal level, 25 genera showed significant differences between the dietary regimens, as shown in the volcano plot (Additional file 1). The microorganisms that differed between the two diets are listed in the supplementary materials (Additional file 2). Those with an average relative abundance greater than one per cent belonged to the following genera: *Prevotella, Falsiporphyromonas, Papallibacter,* and *Parabacteroides* (8.55 vs. 4.39% and 1.37 vs. 2.52 and 0.87% vs. 1.64 and 0.86 vs. 1.62%, CTR vs. SIL, respectively). Only the *Prevotella* genus was more frequent in the CTR group, whereas the other genera were more common in the SIL diet group. The beta diversity was calculated as the Jaccard similarity index and five primary clusters were found (Additional file 3). One group included only subjects fed the CTR diet, whereas the other group included only animals treated with the SIL diet, accounting for approximately 35% of the samples. The remaining microbiota groups are randomly dispersed, implying that nutritional treatment does not appreciably impact the gut microbiota.

## Discussion

Pig meat chains in Italy (over 9 million pigs per year) require mostly heavy animals at slaughter to acquire a sufficient size of the fresh cut (minimum 11 kg) for curing ham production and several fat-wrapped foods. The entire manufacturing process is strictly regulated, and the guidelines were recently updated [12]. Previously, we conducted several trials using WECSs [33, 57] and WPCSs [19] in pigs in the final period of fattening (roughly 90 to 160 kg of BW). We are now encouraged to conduct the current study following the revised standards, which permit a new maximum dietary inclusion of two corn silages (20 and 10% of DM for WECS and WEPS, respectively). Furthermore, our feeding trial considered the final fattening interval from 120 to 180 kg BW, as new guidelines increased the ultimate animal BW (around 180 kg).

The guidelines update underlines an interest in the utilization of silages in animal diets, but other possible concerns must be investigated. A possible limitation is represented by the feeding system commonly adopted for feed distribution in fattening farms and in this sense studies are needed to evaluate the effect of large particle size inclusion.

### Animal performance and characteristics

The experimental diet was created by substituting the entire wheat bran and a portion of the corn meal from the control diet with corn silages to balance the fibre and reach a similar NDF content of about 18% DM. In parallel, to achieve an equal protein content (approximately 14% DM of CP), the soybean dosage had to be slightly increased in the diet containing silages.

The growth performances of the animals were satisfactory, with complete consumption of all the programmed daily rations (refusals less than 5%, without differences between treatments). The rate of growth (750–800 g/d) must be considered high given the advanced interval of growth of animals (from 120 to 180 kg), which is particularly rich in fat with low lean tissues. Recently, published trials [17, 29, 30, 42] reported daily gain values comparable to ours but obtained in earlier growth phases (from 40 to 110 kg). However, the inclusion of corn silages tended to reduce the growth rate by 5–6%. Ma et al. [30] tested different levels of WPCS in the diet of growing pigs and reported that a 10% inclusion level resulted in a substantial decrease in the growth rate between 30 and 60 kg of BW but not a significant decrease between 60 and 100 kg of BW. Friman et al. [17], including approximately 5% of dietary DM with grass silage in its long form, reported a 5% reduction in weight gain.

Pigs fed with silages tended to have a lower performance due to a significant loss in digestibility, particularly in the fibre fraction. Indeed, corn silage fibre is less degradable than bran fibre, and the chemical composition of the diets revealed a greater ADF/NDF ratio in the corn silage diet. Furthermore, it is well known that particle size has a significant impact on digestion, and corn silages are substantially larger than corn and bran meals. Despite these limitations, pigs fed a SIL diet demonstrated a good capacity to digest fibre, which can be related to a well-developed gut, as found by [18, 20] in heavyweight pigs. The silage diet had the same apparent CP digestibility as the control, despite the higher content of soy, which has a greater digestibility than bran (80 vs. 69%, [40]). Pigs fed silages produced faeces with lower DM, and it could be speculated that the coarse diet containing silages provided a more suitable habitat for gut microbial growth. As a result, a greater N influx for microbial protein synthesis resulted in greater metabolic faecal losses and reduced apparent digestibility. Shifting N excretion from urine to faeces is relevant for the environmental impact given the lower volatilization of faecal N compared to that of urinary N.

Considering the limited difference in growth rate, corn silage did not affect animal slaughter traits or fatty acid composition, which confirmed our previous findings in the WECS and WEPS diets [4]. The fatty acid composition of the back fat was similar to that previously described in Italian heavy pigs, with a 40:45:15 ratio between saturated, monounsaturated, and polyunsaturated fats. The only significant change was a decrease in the ω-6/ω-3 ratio of the silage diet, which is a positive trend for consumer health and is mainly due to the decrease (not significant) in linoleic acid.

The gut microbiota of swine can be affected by several endogenous and exogenous factors including the diet [52]. In the present study, no significant variation in the species richness or evenness was achieved for the two dietary groups as demonstrated by alpha diversity indexes, and only marginal differences in a few genera were found. Given the inclusion of corn silages, we expected that dietary treatments impact the cecum microbiota of animals, but only limited differences were observed. Evidently, differences limited to fibre type and particle size of some ingredients (silages) were not enough to reach a clear differentiation in cecum microbiota whereas changes are limited to some microorganisms. For example, the genus Prevotella had the highest relative abundance in the CTR group, which contained more digestible fibre. This genus characterizes the cecum region of the gut environment [56] and includes many species that are mostly saccharolytic and produce short-chain fatty acids [14, 46].

### Prevention of mucosa damage and animal health

The SIL diet substantially affected both stomach development and mucosal integrity. The first consequence was an increase in stomach weight by around 6% (*P* < 0.01). This is consistent with our prior trials, in which the use of 30% WECS or 20% WPCS [33, 57] increased weight ranging from 5 to 10%. This weight increase is a clear reaction to coarse feed in terms of the growth of stomach wall tissues, which is likely required to be stronger to better mix the contents [8]. A second effect was the expansion of the oesophageal area, but despite the statistical relevance level, the effect was modest in terms of magnitude.

The most notable advantage of coarse feeding was a reduction in stomach damage severity, with a low number of cases with higher scores in animals fed coarse materials. The ability of large particles and fibres to prevent gastric mucosa damage has been previously demonstrated [36–38]. Apart from improving gastric health, the provision of forages in long form may have an additional positive effect in terms of the feeding behaviour of animals in comparison to compound feeds not finely ground and/or containing fibrous by-products. Nguyen et al. [39] fed diets containing 10% lucerne hay and reported that pigs apparently rested less and socialized more than control pigs. Friman et al. [17] visually inspected pigs fed with diets containing long-cut grass silages and observed that animals rooted out feed on the floor, increasing the interactions during eating. Coarse ingredients could be an appropriate form of appetitive behaviour (improving searching, rooting, and chewing). We did not directly investigate the aggressiveness and/or nervous state of the animals, and we could not determine whether the use of corn silage affected these aspects.

A method based on hormonal stress markers was used to evaluate the total impact of dietary factors on pig welfare conditions. The hypothalamus–pituitary–adrenal (HPA) axis response system is crucial for maintaining a basal homeostatic state [22, 35] and exposure to repeated or chronic stressors can lead to its dysregulation , resulting in pathophysiological effects [16, 25]. The assessment of cortisol in pig hair samples is a useful tool to evaluate different types of stress factors as reported in previous studies [2, 9, 54]. Moreover, hair hormone analysis provides retrospective information about the endocrine properties of animals over time and is not influenced by acute variations caused by single events or circadian rhythms, providing a measure of the allostatic load [53]. DHEA (S) concentration was assessed because it is released by adrenal glands in response to ACTH (AdrenoCorticoTropic Hormone) as a result of HPA axis activity to comply with its neuroprotective role [25]. It acts as an “anti-stress” steroid, minimizing the negative effects of glucocorticoids such as cortisol [31]. The concentrations of cortisol and DHEA(S) and their ratios at the beginning of the trial did not differ between the two groups indicating a similar allostatic load of the animals involved due to the similar management conditions before the trial began. Similarly, at the end of the trial, the endocrine assets were comparable between the groups with similar concentrations in both hormones investigated. As previously mentioned, cortisol and DHEA(S) have been used in many studies as indicators of stressful conditions, but, to our knowledge, no other study has ever used them as indicators of gastric ulcers in swine. Unfortunately, the two hormones investigated seem to not be strictly related to the gastric condition of animals. Previous studies have tried to relate other welfare to the presence of gastric lesions. Rutherford et al. [47] investigated the behaviour with or without gastric lesions reaching a significant difference in some animal postures but no variation in animal activities. Also, Friman et al. [17] evaluated the effect of pigs’ gastric condition and their behaviour with no significant differences achieved. Considering these results, the direct assessment at the slaughterhouse seems to still be the only method to evaluate gastric condition. To overcome this issue several biomarkers should be considered simultaneously [32], allowing to identify a clear gastric condition indicator.

## Conclusion

The dietary inclusion of corn silages (30% of diet DM) decreased the severity of stomach damage in finishing heavy pigs. However, no appreciable variations in the stress conditions of animals caused by coarse diets were observed, as suggested by the concentrations of cortisol and DHEA(S) in the hair confirming the lack of an appropriate indicator for gastric condition in swine. Based on the performance of the feeding trial, the perspective of feeding heavy pigs corn silage should consider specific agronomic and harvesting techniques (e.g., shorter cuttings and/or anticipated harvesting) to improve digestibility and not reduce the growth rate.

## Supplementary Information

Additional file 1.

Additional file 2.

Additional file 3.

## Data Availability

No datasets were generated or analysed during the current study.
