# Supplementary material for: Dietary inclusion of fibrous corn silages reduces gastric mucosa damage in fattening heavy pigs
Source: Porcine Health Manag. 2024 Nov 22;10:53. doi: 10.1186/s40813-024-00391-9 (PMC11583438; doi:10.1186/s40813-024-00391-9)
Supplement: Supplementary file 1 — Additional file 1. [file 40813_2024_391_MOESM1_ESM.docx]

**Dietary inclusion of fibrous corn silages reduces gastric mucosa damage in fattening heavy pigs**

Spanghero M., Braidot M.^*^, Orioles, M., Sarnataro C., Pividori I., Romanzin A.

Department of Agricultural, Food, Environmental and Animal Sciences, University of Udine, Via Sondrio, 2/A, 33100 Udine, Italy


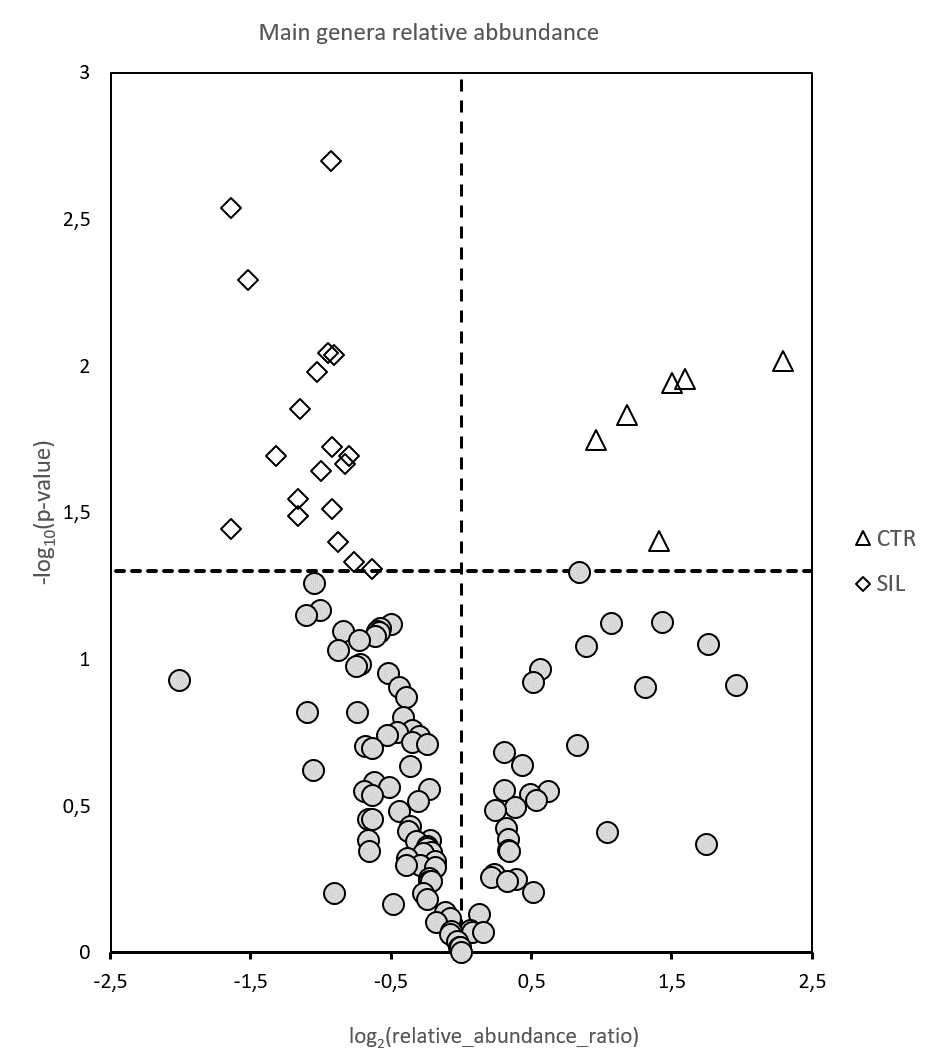
**Supplementary material**

Figure S1. Volcano plot of the main genera found in the gut microbiota of the control diet (CTR) and diet containing silages (SIL). The empty circles represent genera that did not differ significantly (*P* < 0.05) between the two groups.
