# Supplementary material for: Dietary inclusion of fibrous corn silages reduces gastric mucosa damage in fattening heavy pigs
Source: Porcine Health Manag. 2024 Nov 22;10:53. doi: 10.1186/s40813-024-00391-9 (PMC11583438; doi:10.1186/s40813-024-00391-9)
Supplement: Supplementary file 2 — Additional file 2. [file 40813_2024_391_MOESM2_ESM.docx]

**Dietary inclusion of fibrous corn silages reduces gastric mucosa damage in fattening heavy pigs**

Spanghero M., Braidot M.^*^, Orioles, M., Sarnataro C., Pividori I., Romanzin A.

Department of Agricultural, Food, Environmental and Animal Sciences, University of Udine, Via Sondrio, 2/A, 33100 Udine, Italy

**Supplementary material**

Table S1. Relative abundance of bacterial genera in gut microbiota that differ significantly between the two dietary groups.

| Genera, as % of total | DIETS^1^ | | RMSE | p-value |
| --- | --- | --- | --- | --- |
|  | CTR | SIL |  |  |
| Prevotella | 8.555 | 4.394 | 5.868 | 0.018 |
| Falsiporphyromonas | 1.369 | 2.523 | 1.888 | 0.040 |
| Papillibacter | 0.868 | 1.643 | 1.220 | 0.031 |
| Parabacteroides | 0.858 | 1.624 | 1.191 | 0.019 |
| Paraprevotella | 0.699 | 1.240 | 0.900 | 0.022 |
| Anaerophaga | 0.489 | 1.094 | 0.813 | 0.032 |
| Marinilabilia | 0.416 | 0.706 | 0.567 | 0.047 |
| Saccharofermentans | 0.330 | 0.660 | 0.497 | 0.023 |
| Dysgonomonas | 0.219 | 0.486 | 0.345 | 0.014 |
| Streptococcus | 0.365 | 0.074 | 0.206 | 0.010 |
| Christensenella | 0.159 | 0.276 | 0.216 | 0.020 |
| Veillonella | 0.309 | 0.102 | 0.196 | 0.011 |
| Eisenbergiella | 0.122 | 0.273 | 0.178 | 0.028 |
| Anaerobranca | 0.277 | 0.104 | 0.196 | 0.040 |
| Mucilaginibacter | 0.127 | 0.238 | 0.176 | 0.009 |
| Hydrogenibacillus | 0.066 | 0.205 | 0.143 | 0.036 |
| Bulleidia | 0.117 | 0.041 | 0.078 | 0.011 |
| Rikenella | 0.024 | 0.061 | 0.043 | 0.020 |
| Odoribacter | 0.032 | 0.050 | 0.037 | 0.049 |
| Mitsuokella | 0.057 | 0.025 | 0.041 | 0.015 |
| Brassicibacter | 0.025 | 0.049 | 0.036 | 0.009 |
| Sporobacter | 0.015 | 0.047 | 0.031 | 0.003 |
| Cellulosilyticum | 0.019 | 0.036 | 0.026 | 0.002 |
| Clostridium_XII | 0.013 | 0.036 | 0.025 | 0.005 |
| Desulfotomaculum | 0.007 | 0.014 | 0.010 | 0.011 |

^1^ CTR= control diet with 0% corn silages; SIL= diet containing whole ear corn silage and whole plant corn silage (20 and 10 % on DM basis, respectively).
