# Supplementary material for: Dietary inclusion of fibrous corn silages reduces gastric mucosa damage in fattening heavy pigs
Source: Porcine Health Manag. 2024 Nov 22;10:53. doi: 10.1186/s40813-024-00391-9 (PMC11583438; doi:10.1186/s40813-024-00391-9)
Supplement: Supplementary file 3 — Additional file 3. [file 40813_2024_391_MOESM3_ESM.docx]

**Dietary inclusion of fibrous corn silages reduces gastric mucosa damage in fattening heavy pigs**

Spanghero M., Braidot M.^*^, Orioles, M., Sarnataro C., Pividori I., Romanzin A.

Department of Agricultural, Food, Environmental and Animal Sciences, University of Udine, Via Sondrio, 2/A, 33100 Udine, Italy


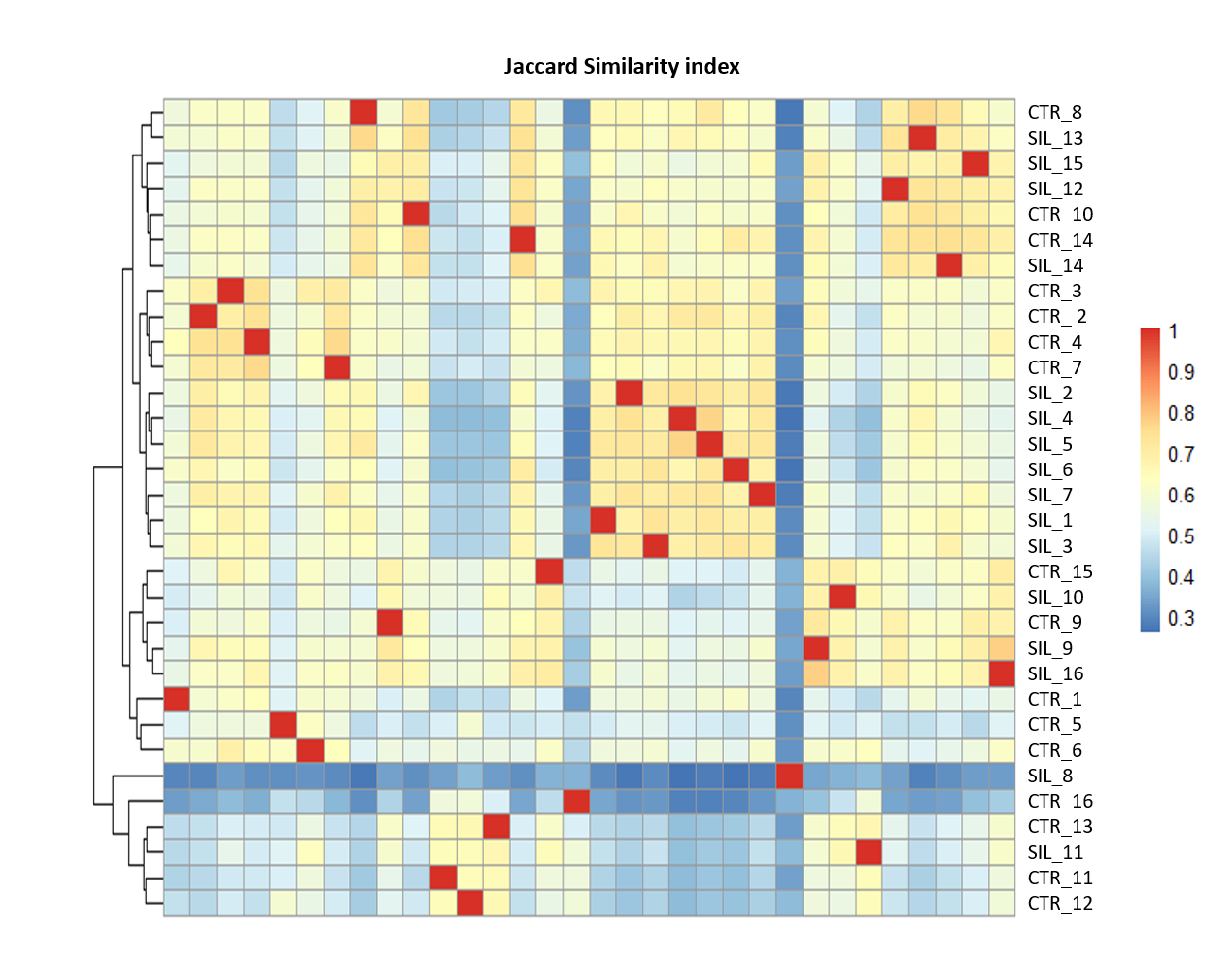
**Supplementary material**

Figure S2. The Jaccard similarity index (beta diversity) calculated for samples of gut microbiota collected from subjects fed with the control diet (CTR) or the silage diet (SIL). A score equal to 1 represents 100% similarity between samples.
